# Neonatal Intensive Care Admissions and Outcomes in Malta from 2019 to 2022—A Retrospective Observational Study

**DOI:** 10.3390/children13040532

**Published:** 2026-04-11

**Authors:** Nadine Anne De Battista, Alexander Attard Littschwager, Clarissa Sciberras, Rebecca Shaw, Ryan Farrugia, Minesh Khashu

**Affiliations:** 1 Department of Child and Adolescent Health, Mater Dei Hospital, MSD 2090 Msida, Malta; nadine-anne.de-battista@gov.mt (N.A.D.B.); alexander.attard-littschwager@gov.mt (A.A.L.); clarissa.sciberras@gov.mt (C.S.); rebecca.shaw@gov.mt (R.S.); ryan.farrugia@gov.mt (R.F.); 2 Department of Neonatology, University Hospitals Dorset NHS Foundation Trust, Bournemouth BH21 3PF, UK

**Keywords:** neonatal care, outcomes, intensive care, morbidity

## Abstract

**Highlights:**

**What are the main findings?**

- NICU admission rates remained consistent before and after the COVID-19 pandemic. A significant proportion of admissions involved term infants, underscoring that neonatal intensive care is frequently required for conditions beyond prematurity.
- Respiratory distress syndrome was the predominant indication for NICU admission; however, surfactant administration rates were relatively low.
- Enteral nutrition practice resulted in delayed attainment of full enteral nutrition compared to international standards. The exclusive breastfeeding rate was below the European Union average.
- Sepsis and central line-associated bloodstream infection (CLABSI) rates were low, suggesting robust infection prevention and control measures.

**What is the implication of the main finding?**

- The Maltese NICU demonstrates overall service stability and effective infection prevention, but opportunities exist to optimize care, especially in respiratory management and nutritional support.
- Targeted quality improvement initiatives, such as adopting standardized feeding protocols and evaluating less-invasive surfactant administration, could help align outcomes with international standards.
- This study highlights the lack of appropriate neonatal data collection in Malta. Establishing a prospective, standardized neonatal database will enable comprehensive data capture, inclusion of high-risk infants, and more robust monitoring of outcomes. Future studies incorporating prospective data collection and including maternal demographics and socioeconomic variables will enhance outcome monitoring to further guide evidence-based improvements in neonatal care.

**Abstract:**

Objective: This retrospective observational four-year review (2019–2022) evaluated neonatal admissions to Malta’s NICU and their outcomes. Study Design: Data from neonates up to 28 days meeting NICU admission criteria with available EMRs were analyzed, focusing on demographic data such as gestation and birth weight, need for resuscitation at birth, admission reasons, and outcomes related to nutrition, respiratory support, congenital anomalies, prematurity-related complications, phototherapy, and infection. Results: Total admissions numbered 1303 (7.3% of total births), out of which 1234 had available electronic medical records and were included in the final analysis. The main reasons for admission were respiratory distress syndrome (27.7%), transient tachypnoea (16.3%), and sepsis (13.5%). Among preterm infants, conditions related to prematurity were observed at expected frequencies and are reported descriptively. Feeding practice resulted in delayed attainment of full enteral nutrition compared to international standards, with an exclusive breastfeeding rate below the EU average. Sepsis and CLABSI rates were low, indicative of robust infection prevention and control measures. Conclusions: This study provides a descriptive overview of NICU admissions and outcomes stratified by gestational age at a single tertiary center in Malta, and highlights areas for improvement. The findings highlight expected patterns of prematurity-related morbidity and differences in clinical management, particularly in nutritional and respiratory support. Future prospective studies incorporating standardized data collection and detailed maternal and demographic variables are needed to better inform neonatal care and service planning.

## 1. Introduction

The neonatal period constitutes a critical window of physiological vulnerability, with substantial evidence demonstrating that optimal healthcare delivery during the first 48 h postnatally is essential for survival and favorable long-term outcomes. Neonatal survival and long-term quality of life depend on the effective prevention and management of conditions such as infections, perinatal asphyxia, congenital anomalies, prematurity, and low birth weight. Over the past six decades, significant advances in neonatal medicine have resulted in increased survival rates and a concomitant rise in admissions to neonatal intensive care units (NICUs) [1]. Neonatal mortality remains the predominant contributor to deaths among children under five years of age [1,2].

Moreover, admissions to neonatal intensive care units impose substantial demands on healthcare resources and entail significant social and financial implications for families [3]. Optimizing neonatal outcomes—defined by decreased mortality and morbidity and improved long-term quality of life—necessitates timely, evidence-based interventions to identify risk factors, prevent complications, and manage treatable conditions. Systematic monitoring of admission rates, management strategies, and patient outcomes is essential to achieve these objectives.

Malta, a small island nation with an estimated population of approximately 530,000 in 2023 and a birth rate of 9.7 per 1000 inhabitants, is served by a single primary tertiary care facility, Mater Dei Hospital [4]. This institution comprises a combined neonatal and pediatric intensive care unit (NPICU) that delivers specialized care for children up to three years of age, while patients aged three years or older are managed in the adult intensive care unit. The present study aims to describe admission patterns, management approaches, and clinical outcomes for neonates up to 28 days of age admitted to the NPICU between 2019 and 2022, with comparisons to international centers.

## 2. Materials and Methods

Ethical approval for this study was obtained from the Chairperson of the Child and Adolescent Health Department, the Chief Executive Officer, and the Data Protection Office at Mater Dei Hospital, Malta. This retrospective analysis utilized electronically maintained hospital records for neonates up to 28 days of age admitted to the NICU between 1 January 2019 and 31 December 2022. Owing to privacy and confidentiality constraints, the dataset is not publicly available.

### 2.1. Study Population, Inclusion and Exclusion Criteria

Inclusion criteria:

- Neonates admitted to the NICU up to 28 days of age between 1 January 2019 and 31 December 2022.
- Available electronic medical records fulfilling NICU admission criteria as outlined in Table 1 below.

Exclusion Criteria:

- Neonates admitted to the NICU after 28 days of life were excluded from the study cohort.
- Neonates for whom electronic medical records were unavailable were also excluded from analysis.

### 2.2. Data Collection and Definitions

This retrospective study relied on routinely collected electronic medical records inputted on discharge, which were not originally designed for research purposes. Consequently, data completeness and variable definitions were dependent on non-standardized documentation practices. Data collection was performed using a structured proforma designed to capture comprehensive demographic and clinical variables, including:

- Infant characteristics: Sex, gestational age, classification as appropriate/large/small for gestational age, resuscitation at birth, and Apgar scores.
- Admission details: Primary diagnosis or indication for admission; nutritional support modalities (expressed breast milk, preterm formula, total parenteral nutrition); respiratory support strategies (high-flow nasal cannula, continuous positive airway pressure [CPAP], bilevel positive airway pressure [BiPAP], invasive intubation); administration of surfactant; and implementation of therapeutic hypothermia.
- Clinical outcomes and comorbidities: Congenital heart disease, patent ductus arteriosus management, retinopathy of prematurity, necrotizing enterocolitis (medical vs. surgical), hyperbilirubinemia management (phototherapy/exchange transfusion), cranial imaging findings, hydrocephalus, chronic lung disease, postnatal dexamethasone, congenital anomalies, surgical interventions, microbiology results (blood, urine, CSF cultures), and hearing assessment.

Given the study’s retrospective design and potential variability in clinical documentation, standardized case definitions were established prior to data extraction. Diagnostic categories, particularly for respiratory conditions such as transient tachypnoea of the newborn (TTN) and respiratory distress syndrome (RDS), were delineated using uniform clinical criteria. These criteria encompassed gestational age, timing of symptom onset, clinical presentation, radiographic features, and the requirement for respiratory support or surfactant therapy. For the purposes of this study, RDS was used predominantly in infants with characteristic ground-glass opacities on chest radiography and significant respiratory distress manifesting shortly after birth. TTN was defined as persistent mild to moderate tachypnoea beyond six hours of life due to delayed clearance of pulmonary fluid, with normal chest imaging and typically transient oxygen requirements.

Where appropriate, variables were aggregated into broader categories to facilitate comparability. Ambiguous cases were assigned to the most consistent and clinically relevant grouping. Imputation of missing or indeterminate data was not performed. This harmonization process ensured consistent classification of each infant, minimized misclassification bias, and enabled meaningful descriptive analysis across the cohort.

### 2.3. Statistical Analysis

Statistical analyses were performed using Microsoft Excel 2024 Version. Continuous variables are reported as mean ± standard deviation (SD) or median and interquartile range (IQR), as appropriate based on data distribution. Categorical variables are summarized as frequencies and percentages. Comparisons between preterm and term infants were conducted using the χ^2^ or Fisher’s exact test for categorical variables, and the independent *t*-test or Mann–Whitney U test for continuous variables. Linear and logistic regression models were employed, where applicable, to examine associations between clinical variables and outcomes. Statistical significance was defined as a two-tailed *p*-value of <0.05.

To evaluate potential shifts in NICU admission patterns associated with the COVID-19 pandemic, data were stratified into pre-pandemic (2019–2020) and post-pandemic (2021–2022) periods. Comparative analyses were performed using appropriate statistical methods, with adjustments for temporal trends in the number of live births during the study period.

### 2.4. Justification and Handling of Missing Data

Infants without available electronic medical records were excluded from the analysis. Due to the absence of a standardized, centralized data collection system in Malta, some variables were incomplete or missing for some patients. While this limited the comprehensiveness of some analyses, it also reflects the current state of the country’s health data infrastructure, highlighting the need for systematic data collection to support evidence-based patient care. For deceased neonates, EMRs were generally unavailable, as these cases typically lack electronic medical records, with paper records being stored offsite. Accessing these paper-based records would have required parental consent and special permissions, which were not feasible within the timeframe of this study. Consequently, only general information available via the hospital registry could be analyzed for these patients.

**Figure 1:**
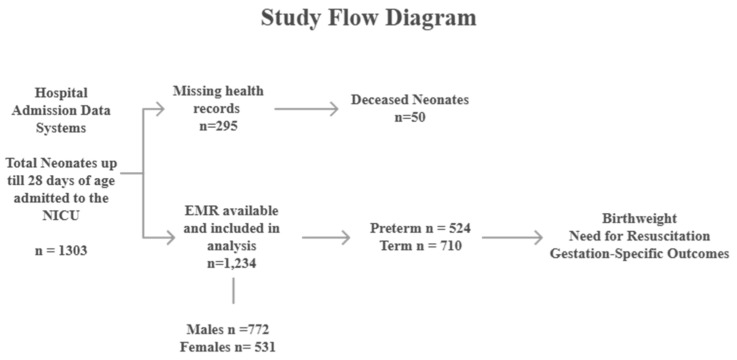
Flow diagram demonstrating the methodological approach employed for participant selection.

For included infants with available electronic medical records, these were analyzed using all available information. The methodological approach is further illustrated in the accompanying flow diagram (Figure 1), which outlines the process of participant selection.

## 3. Results

### 3.1. General Demographics and Admission Trends

The findings presented reflect only the subset of neonates with sufficiently complete electronic medical records available for analysis. A total of 1303 neonates were admitted between January 2019 and December 2022, comprising 772 males and 531 females. Linear regression analysis of admissions over this period yielded a *p*-value of 0.0094, indicating a significant decreasing trend in neonatal admissions across these years.

A total of 50 admitted neonates (31 females, 19 males) passed away during the study period; all had been admitted immediately after delivery. The indication for admission was documented as prematurity in seven cases, congenital heart disease in two cases, and diaphragmatic hernia in one case, while it was not reported for the remaining cases. Two deaths occurred within the first 24 h of life, 18 within the first 72 h, an additional six within the first 7 days, and 12 up to 28 days of age. The remaining deaths occurred beyond the neonatal period. The mean age at death was 32 days. Notably, substantial gaps in mortality-related data were identified, representing a significant limitation that warrants urgent attention and improved data collection practices.

Annual NICU mortality rates demonstrated notable variation over the four-year period analyzed. In 2019, the mortality rate was 5.5% (*n* = 21), which subsequently decreased to 2.39% (*n* = 8) in 2020 and remained relatively stable at 2.6% (*n* = 8) in 2021. However, an increase was observed in 2022, with the mortality rate rising to 4.68% (*n* = 13). A chi-square test of independence was performed to compare NICU mortality across the four years. Although mortality decreased from 2019 to 2020 and then increased again in 2022, the differences were not statistically significant (χ^2^(3) = 5.29, *p* = 0.15). This suggests that the observed variations in mortality rates may be due to random fluctuation rather than meaningful changes over time. Linear regression analysis was performed using 2019 as the reference year. The odds of NICU mortality were significantly lower in 2020 (OR 0.42, 95% CI 0.18–0.98, *p* = 0.045). A similar reduction was observed in 2021, although this did not reach statistical significance (OR 0.45, 95% CI 0.19–1.05, *p* = 0.064). In 2022, mortality was not significantly different from 2019 (OR 0.84, 95% CI 0.41–1.72, *p* = 0.63). These findings suggest a transient reduction in mortality in 2020 that was not maintained in subsequent years.

**Figure 2:**
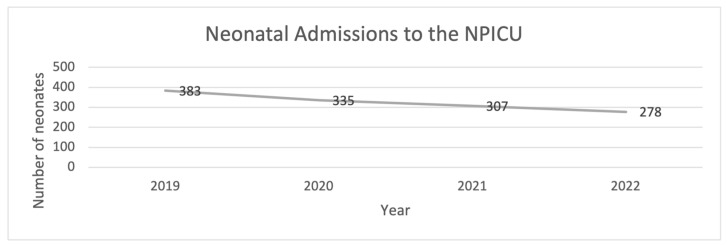
The number of NICU neonatal admissions from 2019 to 2022.

A comparative analysis of admissions pre-COVID (2019) and post-COVID (2020–2022) using the Mann–Whitney U test showed a *p*-value of 0.5, suggesting no statistically significant difference between these periods. The distribution of admissions by year is illustrated in Figure 2.

A total of 295 neonates (22.6% of all admissions), including all recorded fatalities, lacked electronic medical records and were consequently excluded from the analysis. Among these, 53 neonates were documented as deceased. As a result, mortality data could not be assessed within the analyzed cohort, as all recorded deaths occurred among excluded cases with incomplete records. No clinical or demographic data were available for infants excluded from the analysis, precluding direct comparison between included and excluded groups. Thus, the analyzed dataset comprises a subset of NICU admissions with comprehensive documentation, rather than the entire admission population.

A cohort of 1234 neonates, aged up to 28 days, had comprehensive electronic medical records, enabling detailed analysis of demographic characteristics and clinical outcomes in the neonatal intensive care unit (NICU).

Table 2 summarizes the demographic and clinical characteristics of neonates admitted to the NICU during the study period. Among these, 524 were preterm (gestational age up to 36 weeks and 6 days at admission), while 710 were term infants (gestational age 37 weeks to 28 days postnatal at admission).

The majority of neonatal admissions (approximately 70%) occurred among infants with a gestational age between 33 and 38 weeks. Very preterm infants, defined as those born between 24 and 27^+6^ weeks of gestation, constituted a small proportion of the cohort (~2.5%). A considerable subset of neonates (~13.6%) had missing or unavailable gestational age data, which likely correspond to term births.

A total of 56.8% of neonates weighed more than 2500 g, while 29.8% weighed between 1500 and 2500 g. Infants with low birth weight (<1500 g) accounted for 8.9% of the cohort.

A total of 73.4% were classified as appropriate for gestational age, while 18.4% were considered small for gestational age. Statistical analysis using the chi-square test demonstrated a highly significant association between gestational age and birth weight (*p* < 0.0001).

### 3.2. Resuscitation at Birth

A total of 90.3% of infants did not require resuscitation at birth. Chi-square analysis demonstrated a strong association between gestational age and the need for resuscitation (*p* < 0.001), as well as between birth weight and resuscitation (*p* < 0.001). Logistic regression modeling, conducted to estimate the probability of resuscitation based on gestational age and birth weight categories, identified significant negative coefficients for gestational age. This finding indicates that increasing gestational age is associated with reduced odds of requiring resuscitation relative to the reference category (24–27^+6^ weeks). In contrast, birth weight categories did not demonstrate statistically significant associations with resuscitation requirements (*p* > 0.05). Additionally, there was no significant difference in resuscitation rates between neonates classified as small for gestational age (SGA) and those appropriate for gestational age, as indicated by a chi-square value of 0.31 and a *p*-value of 0.86.

### 3.3. Reasons for NICU Admission

**Figure 3:**
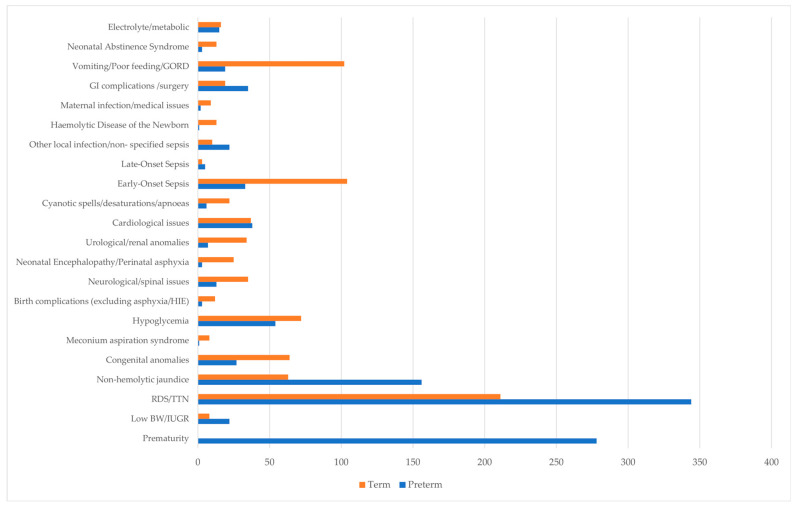
Indications for admission to the neonatal intensive care unit.

The indications for admission to the neonatal intensive care unit are demonstrated in Figure 3.

### 3.4. Nutrition Practices

A chi-square test was employed to assess whether the distribution of feeding modalities—exclusive expressed breast milk (EBM), exclusive preterm formula (PTF), total parenteral nutrition (TPN), and mixed feeding—differed significantly between preterm and term infants. The test yielded a chi-square statistic of 314.91 with three degrees of freedom and a *p*-value of 5.89 × 10^−68^, far exceeding conventional thresholds for statistical significance (e.g., *p* < 0.05). These findings indicate a statistically significant disparity in feeding type distribution: PTF and TPN usage were markedly more prevalent among preterm infants, whereas exclusive EBM was more frequently observed in term infants.

A two-sample *t*-test was conducted to compare the mean time (in days) required to achieve full enteral feeding between preterm and term neonates. Preterm infants required a mean of 11.03 days, whereas term infants achieved full enteral feeding in a mean of 5.89 days. The *t*-statistic was 7.49, with a *p*-value of 3.88 × 10^−13^, indicating a highly significant difference. These results demonstrate that preterm neonates require substantially more time to reach full enteral feeding compared to their term counterparts.

Additionally, a one-way analysis of variance (ANOVA) was performed to evaluate differences in the time required to achieve full enteral feeding across birth weight categories. The ANOVA yielded an F-statistic of 84.55 and a *p*-value of 3.94 × 10^−45^, indicating significant differences among the groups. The mean times to achieve full enteral feeding were as follows: 21.01 days for very low birth weight (<1.5 kg), 7.48 days for low birth weight (<2.5 kg), 6.02 days for normal birth weight (2.5–4 kg), and 7.38 days for high birth weight (>4 kg). These findings demonstrate that neonates with very low birth weight require a substantially longer time to achieve enteral feeding than those with normal birth weight. A significant difference was also observed between the normal- and high-birth weight groups.

### 3.5. Associated Complications for Admitted Neonates

Complete morbidity data were available for 1075 infants, representing 86.9% of the cohort. Hypoxic–ischemic encephalopathy requiring therapeutic hypothermia was reported in nine term infants (8.37 per 1000 births). Several other low-frequency events were observed: ventricular dilatation or hydrocephalus occurred in 10 preterm and two term infants, of whom only three preterm infants required surgical intervention; congenital anomalies were noted in 18 preterm and 29 term infants; and any surgical intervention was performed in 30 preterm and 28 term infants. Due to the low counts, statistical comparisons were not conducted for these variables. Hyperbilirubinemia requiring phototherapy was significantly more common in preterm infants than in term infants (295/528 [55.9%] vs. 99/547 [18.1%], *p* < 0.001). In contrast, abnormal hearing screen results were observed in 73 preterm infants (13.8%) and 62 term infants (11.3%), with no statistically significant difference between groups (*p* = 0.43). These findings indicate that preterm infants are at substantially higher risk of hyperbilirubinemia requiring intervention, whilst hearing abnormalities do not differ significantly by gestational age. Table 3 below demonstrates complications and morbidities observed for the preterm cohort.

### 3.6. Respiratory Support

**Figure 4:**
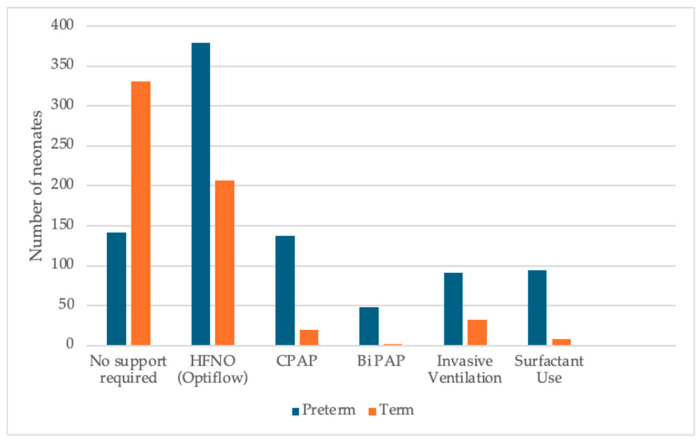
Respiratory support use in neonates up till 28 days of age.

**Figure 5:**
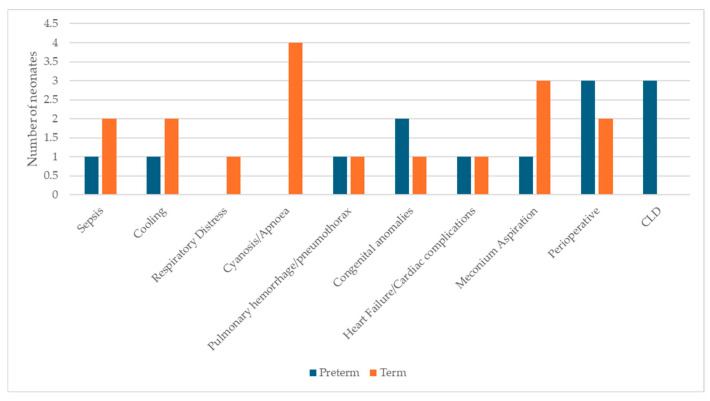
Indications for respiratory support.

Figure 4 and Figure 5 illustrate the spectrum of respiratory support modalities and their respective clinical indications among neonates admitted to the NICU. Statistical analysis revealed a highly significant difference in respiratory support requirements between preterm and term infants (*p* < 0.0001).

### 3.7. Cardiac Morbidity

**Figure 6:**
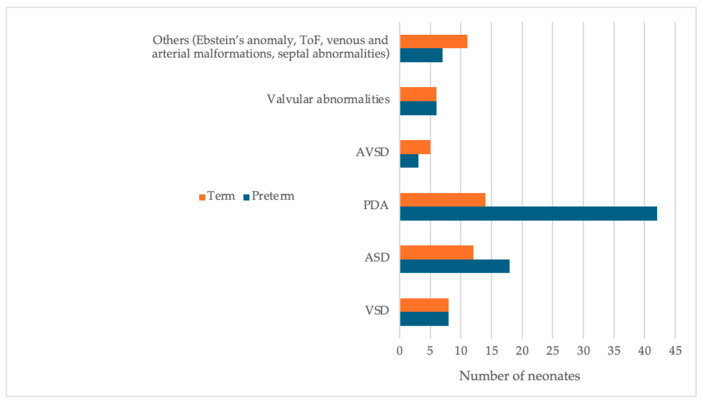
Congenital heart disease and ECHO findings for admitted neonates.

Figure 6 depicts the data collected on congenital heart disease and echocardiographic findings in neonates admitted to the NICU. A statistically significant difference in echocardiographic findings was observed between the two groups (*p*-value = 0.0267).

### 3.8. Sepsis

Table 4 summarizes the rates of positive culture results among neonates admitted to the NICU, as well as the mean age at which the first positive culture was identified. Statistical analysis revealed no significant difference in culture positivity rates between preterm and term infants. However, a highly significant difference was observed in the mean age at first positive culture (*p* < 0.0001).

## 4. Discussion

This retrospective observational study aimed to provide a comprehensive characterization of the demographic attributes, clinical management paradigms, and outcomes of neonates admitted to the NICU at Mater Dei Hospital, the sole tertiary center in Malta. Emphasis was placed on outcome analyses for both term and preterm populations. However, interpretation of these findings is substantially constrained by inherent data limitations. The analytic cohort was restricted to neonates with available electronic documentation, while all mortality cases were excluded due to absent records. Consequently, the reported findings likely underestimate the true burden of neonatal morbidity and mortality within the unit.

These limitations highlight the critical need to develop and implement a comprehensive, real-time neonatal data registry. Such an infrastructure would enhance data completeness and integrity, strengthen clinical governance and audit processes, and ensure systematic inclusion of high-risk neonates—particularly those with severe morbidity or fatal outcomes—who are frequently underrepresented in retrospective analyses.

Furthermore, adopting a prospective data acquisition framework would facilitate longitudinal follow-up, enabling robust evaluation of both short- and long-term outcomes, including neurodevelopmental trajectories. This approach would significantly enhance methodological rigor and provide a stronger evidentiary basis for clinical decision-making and service planning.

Given the retrospective study design and the absence of key demographic and clinical confounders, the observed associations should be interpreted as hypothesis-generating rather than indicative of causal relationships or definitive temporal changes in clinical practice. Trends in management over time must therefore be interpreted with caution, as the dataset is derived from non-standardized retrospective documentation. Apparent variations in clinical practices—such as respiratory support strategies or nutritional interventions—may, at least in part, reflect inconsistencies in documentation or classification rather than true evolution in care delivery.

Despite efforts to standardize variable definitions and harmonize clinical terminology, the absence of a prospective and uniformly applied data collection system limits the precision with which temporal trends can be reliably assessed.

### 4.1. Mortality Rates, Admission Rates and Demographic Characteristics

NICU admission rates remained relatively stable throughout the study period, with only a modest decline of approximately 5%. This stability may be partially attributable to recent modifications in admission criteria, including the adoption of higher umbilical cord pH thresholds. Additionally, improvements in antenatal diagnostic accuracy may have facilitated the in utero transfer of high-risk pregnancies to tertiary centers abroad.

In this study, NICU mortality demonstrated year-to-year variability without a statistically significant overall trend. Although a marked reduction in mortality was observed in 2020, this improvement was not sustained in subsequent years. Logistic regression analysis confirmed significantly lower odds of mortality in 2020 compared to 2019, suggesting a transient improvement in outcomes during that period. This finding may reflect changes in clinical protocols, improved infection control measures, or alterations in patient case mix, particularly in the context of the COVID-19 pandemic, which influenced healthcare systems globally. However, the rise in mortality observed in 2022 indicates that these improvements were not maintained, potentially due to normalization of admission patterns, increased patient acuity, or resource constraints. The absence of a consistent linear trend suggests that fluctuations in mortality are more likely attributable to temporal and systemic factors rather than sustained changes in quality of care. This analysis was limited by the lack of adjustment for important clinical variables such as gestational age, birth weight, and severity of illness, which may have influenced mortality outcomes, and continues to highlight the importance of continuous monitoring of NICU outcomes and the need for further studies incorporating risk-adjusted analyses to better understand the underlying drivers of mortality.

The principal indications for NICU admission included respiratory issues, prematurity, and hyperbilirubinemia. Although gestational age remains a primary determinant of NICU requirement, term neonates with appropriate birth weight may nonetheless necessitate intensive care. In this cohort, term infants constituted a greater proportion of admissions than preterm infants, with the majority born between 37 and 38^+6^ weeks’ gestation. This observation is consistent with prior research indicating that approximately 50% of NICU admissions in 38 U.S. states involved neonates born after 37 weeks of gestation [5].

Preterm neonates accounted for 42.7% of admissions, of whom 27.8% were classified as late preterm, representing approximately two-thirds of the preterm subgroup. This distribution aligns with global epidemiological patterns and likely reflects the heightened vulnerability of this subgroup [6]. Low birth weight (<2.5 kg) was observed in 38.1% of admissions, including 9% categorized as very low birth weight (<1.5 kg), consistent with the existing literature [6]. Notably, 73% of admitted neonates were of appropriate weight for gestational age.

Resuscitative interventions at birth were required in 8.2% of cases, predominantly involving respiratory support. This finding is consistent with international estimates indicating that approximately 10% of neonates require some degree of respiratory assistance at delivery, with around 1% requiring advanced resuscitation measures [7].

Interpretation of these findings must consider evolving demographic trends within the Maltese population, including increasing maternal age, rising immigration, and greater ethnic diversity. These factors represent potential unmeasured confounders that could not be incorporated into the present analysis due to data limitations, thereby constraining the ability to fully account for population-level influences on NICU admission patterns and outcomes.

### 4.2. Prematurity-Related Morbidity

Prematurity is strongly associated with a spectrum of complications, including intraventricular hemorrhage (IVH), periventricular leukomalacia (PVL), retinopathy of prematurity (ROP), and necrotizing enterocolitis (NEC). Among infants born before 30 weeks’ gestation, 26.25% developed ROP, with 19% requiring laser intervention. This incidence falls within the lower range of global reports, which vary from 11% to 65% across European NICUs [8,9].

In our study, we report incidence rates for IVH, PVL, ventricular dilatation, and hydrocephalus of 3.6%, 4.2%, and 2.3%, respectively. While comprehensive European prevalence data remain limited, studies from other regions report substantially higher rates, ranging from 20% to 50%, depending on population characteristics and institutional practices [10]. The comparatively lower incidence observed in this cohort may reflect the limited proportion of extremely preterm and very-low-birth-weight infants, given that gestational age and birth weight are key determinants of IVH risk.

NEC, a severe gastrointestinal pathology predominantly affecting preterm neonates, was observed in 5.9% of the preterm cohort (2.9% of total admissions). Surgical intervention was required in 29% of cases, consistent with previously reported estimates [11]. The incidence of NEC in very-low-birth-weight infants is reported to range from 5% to 10%, with approximately 30% requiring surgical management and mortality rates approaching 50%. Multi-center studies estimate that NEC affects up to 5% of NICU admissions [12,13]

### 4.3. Nutritional Practices

Optimal nutritional provision in the NICU is essential for improving long-term neurodevelopmental outcomes [14]. In this cohort, expressed breast milk was administered to 41.9% of preterm and 35.5% of term neonates, yielding an overall rate of 38.7%, with exclusive breastfeeding observed in only 8.8% of cases. These findings are lower than those reported in other settings, where exclusive breastfeeding rates at discharge have been documented at 52% in Malaysian units and 32% in UK units [15,16,17].

Formula feeding was observed in 30.9% of preterm infants and 0.4% of term neonates. Additionally, 29.5% of preterm infants required total parenteral nutrition (TPN), compared with 1.5% of term infants, accounting for 15.3% of total admissions. These figures are broadly comparable to those reported in other countries, including the UK [18].

Differences in feeding strategies between preterm and term infants are consistent with prior findings from the same unit, which demonstrated predominant use of mixed feeding regimens, with formula frequently comprising a substantial proportion of intake [19]. Birth weight and gestational age were key determinants of feeding modality, with infants of lower gestational age more likely to receive breast milk.

The mean time to achieve full enteral feeding was significantly longer in preterm neonates (11.03 days) than in term infants (5.89 days). These findings align with international data suggesting that full enteral feeding is typically achieved within 11 to 15 days in preterm infants managed under structured feeding protocols [20,21]. For very-low-birth-weight infants, a target of achieving full enteral feeding within 14 days is generally recommended [22].

In our cohort, very-low-birth-weight infants required a mean of 21.01 days to reach full enteral feeding, exceeding benchmarks reported in studies using accelerated feeding protocols, in which full feeds may be achieved in approximately 8 days [23]. Implementation of standardized feeding strategies has been shown to reduce time to full feeds and decrease reliance on parenteral nutrition [23,24]. These delays likely reflect a combination of conservative clinical practices, decision-making variability, and the absence of standardized protocols. Differences in patient case mix and institutional risk tolerance may also contribute. However, the absence of detailed data on feeding protocols limits further interpretation. These findings highlight a clear opportunity for targeted quality improvement initiatives to optimize enteral feeding progression.

### 4.4. Respiratory Management

Respiratory distress syndrome remained the leading indication for NICU admission, with 73% of preterm neonates requiring respiratory support, although only 17% required invasive ventilation. These findings are consistent with global trends reflecting advances in antenatal corticosteroid use and non-invasive ventilation strategies [25,26].

Surfactant therapy was administered to 17.8% of preterm infants and 1.5% of term infants. Among neonates born between 22^+0^ and 32^+6^ weeks’ gestation, 37.14% received surfactant, which is lower than that reported in Europe and tertiary NICU practice patterns [26,27].

In late preterm infants (34–36 weeks), surfactant administration is reported in approximately 10% to 20% of cases [28]; however, only 1.43% of infants in this gestational range received treatment in this cohort. This comparatively low utilization warrants further evaluation, particularly considering evolving practices such as less invasive surfactant administration (LISA), which have improved the safety profile of surfactant delivery.

Chronic lung disease was identified in 7.4% of preterm infants, with 5.1% receiving postnatal dexamethasone therapy. These findings are consistent with reported corticosteroid use rates of approximately 5% in similar populations [27,29,30,31]. 

The prevalence of bronchopulmonary dysplasia varies widely across studies, ranging from 5% to 75% [30], with higher rates reported in North American cohorts [30,31].

Interpretation of respiratory management patterns is limited by the lack of granular clinical data, including disease severity and timing of interventions, underscoring the need for prospective data collection.

### 4.5. Sepsis

The prevalence of early-onset sepsis was 12.7%, compared to 0.7% for late-onset sepsis, with an additional 2.98% of cases unclassified. No significant differences in culture positivity were observed between term and preterm groups, although preterm infants demonstrated a higher proportion of positive blood cultures.

The overall blood culture positivity rate was 4.8%, consistent with findings reported in other settings [32,33]. Central line-associated infections were observed in 1.7% of preterm infants and 0.2% of term infants, rates substantially lower than those reported in other NICUs [34].

The mean time to culture positivity was significantly longer in preterm infants (22.88 days) compared to term infants (5.79 days), reflecting prolonged hospitalization and increased exposure to invasive procedures. Reported rates of culture-positive sepsis across European NICUs vary widely, ranging from 12% to 78% depending on study design and population characteristics [35].

### 4.6. Additional Outcomes

Hyperbilirubinemia requiring treatment was observed in 37.3% of neonates, with 55.9% of preterm and 18.1% of term infants requiring phototherapy. These rates exceed those reported in the United Kingdom and are likely influenced by multifactorial factors, including feeding practices and perinatal factors [36]. Perinatal asphyxia was documented in 4.6% of term admissions, exceeding UK estimates of approximately 2.5%. Therapeutic hypothermia was administered in 1.5% of cases, consistent with international data [37]. A significant difference in the prevalence of patent ductus arteriosus was observed between preterm and term infants. Management was relatively conservative, with paracetamol used in 1.3% of cases, NSAIDs in 1.1%, and surgical ligation in 0.4%. These rates are substantially lower than the 45% treatment rate reported in multinational cohorts [38].

As a small island nation with a single tertiary center, Malta faces unique challenges in neonatal care delivery and benchmarking. A recent Nordic study by Norman et al. (2023) that included Iceland—another small island nation with centralized neonatal care—demonstrated that despite significant variations in clinical management practices, such as use of surfactant, survival and major morbidity outcomes were similar with no statistically significant differences [39]. This suggests that different approaches to neonatal care, as observed in our cohort for nutritional and respiratory support practices, may not necessarily indicate suboptimal care but rather reflect context-specific adaptations. Malta’s centralized structure, while limiting opportunities for internal benchmarking, might offer better care coordination and standardization that might be lacking in larger fragmented systems. However, the lack of long-term systematic follow-up data remains a critical gap. This is required to confirm whether our management approaches translate into equivalent developmental and health outcomes. Establishing systematic post-discharge monitoring incorporating neurodevelopmental assessment, nutritional surveillance, and family-centered care would enable Malta to evaluate whether the observed practice variations translate into long-term outcome differences.

## 5. Limitations

This study has several limitations. The relatively short study period limits generalizability. Moreover, a substantial proportion of NICU admissions (22.6%) were excluded due to missing or incomplete records, including all mortality cases, introducing significant selection and survival bias and likely resulting in underestimation of adverse outcomes. The retrospective design relied on non-standardized electronic medical records not originally intended for research purposes. Variability in documentation and clinical terminology introduces potential misclassification bias despite efforts to standardize definitions.

Additionally, the absence of key demographic and clinical variables—including maternal age, ethnicity, socioeconomic status, and obstetric history—precluded adjustment for important confounders. Demographic shifts within the Maltese population, including increased maternal age and immigration, may further influence neonatal outcomes [40,41]. Immigrant populations, particularly women with limited healthcare access, have been shown to experience poorer neonatal outcomes [42]. Furthermore, increasing maternal age, with births to women aged ≥ 40 rising substantially over recent decades, represents an additional influencing factor [43].

These limitations reflect an important structural gap in the current data infrastructure. Despite Malta having a single tertiary neonatal center, there is no comprehensive, standardized, prospectively maintained neonatal database. The inability to retrieve complete outcome data for a substantial proportion of admissions highlights the urgent need for a unified national system capable of capturing detailed, high-quality neonatal data. The absence of systematic post-discharge follow-up data precludes assessment of long-term neurodevelopmental and health outcomes, limiting the ability to evaluate the downstream impact of variations in acute neonatal management.

This study was conducted within a single center over a limited time period, which may restrict generalizability. Although this center provides all tertiary neonatal care nationally, caution is warranted when extrapolating findings to other healthcare settings with different population characteristics and care structures.

Given these limitations, the findings should be interpreted as descriptive and hypothesis-generating. The available data do not permit causal inference or robust assessment of temporal trends in clinical practice.

## 6. Conclusions

This four-year, single-center study provides a descriptive, stratified overview of NICU admissions and outcomes at the sole tertiary center in Malta. A substantial proportion of admissions involved term neonates, underscoring that intensive neonatal care needs extend beyond prematurity. Variability in clinical practices, particularly in the management of nutritional and respiratory distress, highlights opportunities for targeted quality improvement. However, the retrospective design, incomplete data capture, and exclusion of mortality cases substantially limit interpretability, and the findings should therefore be considered descriptive and hypothesis-generating rather than definitive.

These results underscore the need for a prospective, standardized neonatal data registry to ensure comprehensive data capture—particularly for high-risk infants—and to support robust outcome monitoring. Future research incorporating detailed maternal, demographic, and socioeconomic variables will be essential to better elucidate population-level determinants and guide evidence-based improvements in neonatal care.

## Figures and Tables

**Table 1:** NICU admission criteria.

Indication	Details
Gestational Age	All infants below or equal to 34^+6^ weeks of gestation
Birth Weight	Neonates with birth weight < 1.9 kg
Resuscitation Need	Advanced resuscitation at birth beyond inflation breaths and persistent issues despite ventilation breaths; perinatal asphyxia
Respiratory Indications	Distress, need for oxygen support, cyanosis, oxygen saturation instability, meconium aspiration
Cardiovascular Instability	Cardiovascular instability
Complicated Delivery	Complicated delivery, traumatic birth injury with severe cephalhematoma, infants of mothers with complications
Neurological Concerns	Seizures, HIE, altered tone or consciousness, suspected intracranial hemorrhage
Hypoglycemia	Three episodes of hypoglycemia 1.2–2.5 mmol/L recorded on *HemoCue (Ängelholm, Sweden)* venous blood gas despite adequate feeding with need for intravenous dextrose support, any *HemoCue (Ängelholm, Sweden)* and venous blood gas < 1.2 mmol/L or symptomatic hypoglycemia
Persistent Acidosis	pH < 7, BE > −12 mmol/L and lactate > or equal to 7 mmol/L 2 h post-birth
Hydronephrosis	Male babies with antenatally diagnosed bilateral hydronephrosis requiring catheterization or suspected postnatal posterior urethral valves
Inborn Errors of Metabolism	Suspected inborn errors of metabolism
Early-Onset Sepsis	Suspected early-onset sepsis
Feeding Intolerance	Feeding intolerance or significant abdominal distension
Congenital Anomalies	Congenital anomalies requiring surgery (e.g., gastroschisis, diaphragmatic hernia)
Postoperative Monitoring	Need for postoperative monitoring
Thermal Support	Inability to maintain body temperature and persistent need for an incubator or thermal support
Severe Hyperbilirubinemia	Severe hyperbilirubinemia requiring intensive phototherapy and exchange transfusion
Continuous Monitoring	Any newborn requiring continuous monitoring (cardiorespiratory)

**Table 2:** Characteristics of neonates admitted to the neonatal intensive care unit during the study period.

Variables		*n* = 1234	%
Gestational age (weeks)	24–27^+6^	31	2.5
	28–30^+6^	59	4.8
	31–32^+6^	91	7.4
	33–34^+6^	169	13.7
	35–36^+6^	174	14.1
	37–38^+6^	258	20.9
	39–39^+6^	145	11.8
	40^+^	139	11.3
	Not available	168	13.6
Birth weight (g)	<1500	110	8.9
	1500–2500	368	29.8
	>2500	701	56.8
	Not available	55	4.5
Gestational age vs. BW	Appropriate	906	73.4
	Small	227	18.4
	Large	64	5.2
	Not recorded	37	3
Resuscitation at birth	Yes	101	8.2
	No	1114	90.3
	Not recorded	19	1.5

**Table 3:** Complications and morbidities of preterm neonatal admissions.

Variable	Preterm Up to 36^+6^ Weeks (*n* = 528) *n* (%)
**Retinopathy of prematurity**	24 (4.5)
Treated	4 (0.8)
Not treated	20 (3.8)
**Intraventricular hemorrhage**	
None	509 (96.4)
Grade 1	11 (2.1)
Grade 2	5 (0.9)
Grade 3	1 (0.2)
Grade 4	2 (0.4)
**Periventricular leukomalacia**	
None	508 (96.2)
Unilateral	7 (1.3)
Bilateral	13 (2.5)
**Necrotizing enterocolitis**	
None	504 (95.5)
Medical treatment	22 (4.2)
Surgical treatment	9 (1.7)
**Patent ductus arteriosus**	
No treatment	29 (5.5)
Paracetamol	7 (1.3)
NSAIDs	6 (1.1)
Surgical	2 (0.4)

**Table 4:** Positive culture rates for neonates admitted to the NPICU and the mean age of first detectable positive cultures.

Positive Cultures	Preterm (*n* = 528)		Term (*n* = 547)		*p* Value
	*n*	%	*n*	%	0.222
Blood (peripheral)	29	5.5	13	2.4	
Blood (central)	9	1.7	1	0.2	
Urine	1	0.2	1	0.2	
CSF	2	0.4	3	**0.5**	
Mean age at which the culture first turned positive	22.88 days		5.79 days		**<0.0001**

## Data Availability

The data presented in this study are available on request from the corresponding author. Raw original data are unavailable due to privacy and ethical restrictions.

## Abbreviations

The following abbreviations are used in this manuscript:

NICU	Neonatal intensive care unit
CLABSI	Central line-associated bloodstream infection
RDS	Respiratory distress syndrome
TTN	Transient tachypnoea of the newborn
PVL	Periventricular leukomalacia
IVH	Intraventricular hemorrhage
VLBW	Very low birth weight
LBW	Low birth weight
NSAID	Non-Steroidal Anti-Inflammatory Drugs
ROP	Retinopathy of prematurity
EBM	Expressed breastmilk
TPN	Total parenteral nutrition
BPD	Bronchopulmonary dysplasia
CSF	Cerebrospinal fluid
NEC	Necrotizing enterocolitis
SGA	Small for gestational age
PTF	Preterm formula
LISA	Less invasive surfactant administration
